# Anti-Biofilm Effect of Selected Essential Oils and Main Components on Mono- and Polymicrobic Bacterial Cultures

**DOI:** 10.3390/microorganisms7090345

**Published:** 2019-09-12

**Authors:** Erika Beáta Kerekes, Anita Vidács, Miklós Takó, Tamás Petkovits, Csaba Vágvölgyi, Györgyi Horváth, Viktória Lilla Balázs, Judit Krisch

**Affiliations:** 1 Department of Microbiology, Faculty of Science and Informatics, University of Szeged, H-6726 Szeged, Közép fasor 52, Hungary; 2 Institute of Food Engineering, Faculty of Engineering, University of Szeged, H-6724 Szeged, Mars tér 7, Hungary; 3 Department of Pharmacognosy, University of Pécs, H-7624 Pécs, Rókus utca 2, Hungary

**Keywords:** antibacterial activity, biofilm, polymicrobial biofilm, essential oil, food spoilage

## Abstract

Biofilms are surface-associated microbial communities resistant to sanitizers and antimicrobials. Various interactions that can contribute to increased resistance occur between the populations in biofilms. These relationships are the focus of a range of studies dealing with biofilm-associated infections and food spoilage. The present study investigated the effects of cinnamon (*Cinnamomum zeylanicum*), marjoram (*Origanum majorana*), and thyme (*Thymus vulgaris*) essential oils (EOs) and their main components, i.e., trans-cinnamaldehyde, terpinen-4-ol, and thymol, respectively, on single- and dual-species biofilms of *Escherichia coli*, *Listeria monocytogenes*, *Pseudomonas putida,* and *Staphylococcus aureus*. In dual-species biofilms, *L. monocytogenes* was paired with each of the other three bacteria. Minimum inhibitory concentration (MIC) values for the individual bacteria ranged between 0.25 and 20 mg/mL, and trans-cinnamaldehyde and cinnamon showed the highest growth inhibitory effect. Single-species biofilms of *L. monocytogenes*, *P. putida,* and *S. aureus* were inhibited by the tested EOs and their components at sub-lethal concentrations. Scanning electron microscopy images showed that the three-dimensional structure of mature biofilms embedded in the exopolysaccharide matrix disappeared or was limited to micro-colonies with a simplified structure. In most dual-species biofilms, to eliminate living cells from the matrix, concentrations exceeding the MIC determined for individual bacteria were required.

## 1. Introduction

Surfaces in the food industry provide an excellent substrate for the development of biofilms by spoilage and pathogenic bacteria [1]. These biofilms are typically highly persistent and can increase the likelihood of cross-contamination, potentially leading to food deterioration and/or serious food-borne diseases [2,3,4,5].

Biofilms are heterogeneous in vivo, comprising different microorganisms that interact with each other and form a complex multi-species community. Cooperation or competition occurs between the species and it shapes the community by influencing attachment, microcolony formation, and/or resistance to stress conditions [6,7,8,9,10]. Multi-species biofilms may exhibit enhanced fitness and react differently to antimicrobials from monocultures and planktonic cells [11].

Contamination with *Listeria monocytogenes* causes a large number of food poisoning outbreaks annually [12]. According to a study performed by the European Food Safety Authority in 2018, this food-borne pathogen is the leading cause of hospitalization and death in Europe [13]. Listeriosis is frequently associated with fish and fishery products, ready-to-eat salads, and different meat products. Paté, butter, and soft and semi-soft cheeses are also potential carriers of the pathogen [13,14]. Its persistence under different environmental stresses (e.g., low temperature) makes it difficult to eradicate and cross-contamination risk is high [15]. *Staphylococcus aureus*, *Escherichia coli,* and *Pseudomonas putida* strains are also associated with food spoilage and food poisoning outbreaks [16,17]. Moreover, certain food-spoilage bacteria such as *P. putida* can enhance the adhesion, colonization, and biofilm formation of *L. monocytogenes*, whereas others such as *Staphylococcus sciuri* can inhibit it [18,19]. Although the chemical preservatives that are permitted in foods are considered to not cause any side effects, concerns have been raised about the safety of nitrites and sulphites. To date, no conclusive evidence that nitrite is directly carcinogenic has been provided, but at high doses it has been suggested to be a co-carcinogen [20,21].

Against this background, there is increasing demand for healthier products and natural food additives. To meet these demands, researchers have started to examine natural preservatives instead of synthetic ones [22]. Plants are a source of a range of substances with antimicrobial properties, which are promising candidates for the development of new anti-infective agents [23]. Among these, essential oils (EOs) from aromatic and medicinal plants have been a focus of attention in recent decades [24,25,26,27]. In addition, the capacity of some EOs to inhibit biofilm formation in mono- and polymicrobial systems has been documented, suggesting their potential utilization as food preservatives and sanitizing agents [28,29,30]. In this context, targeting polymicrobial cultures with EOs might be effective for reducing the growth and activity of food-related pathogens.

In previous studies, lemon, marjoram, and cinnamon EOs showed inhibitory effects against *E. coli* and *P. putida* biofilms in mixed-culture systems [31,32]. Here the influence of cinnamon, marjoram, and thyme EOs and their major components, namely, trans-cinnamaldehyde, terpinen-4-ol, and thymol, respectively, on the formation of *L. monocytogenes*, *E. coli*, *S. aureus,* and *P. putida* mono- and polymicrobial biofilms was investigated.

## 2. Materials and Methods

### 2.1. Bacterial Strains

The bacterial strains used in this study were provided by the Szeged Microbiological Collection (SZMC). The Gram-positive *L. monocytogenes* SZMC 21307 and *S. aureus* SZMC 110007 and the Gram-negative *E. coli* SZMC 0582 and *P. putida* SZMC 291T were used to form mono- and dual-species biofilms.

For pre-culturing and biofilm formation, tryptic soy broth (TSB) containing (in %) peptone from casein 1.7 (Merck; Budapest, Hungary), peptone from soy meal 0.3 (Oxoid; Hampshire, UK), D(+)-glucose 0.25 (VWR; Debrecen, Hungary), NaCl 0.5 (VWR), and K_2_HPO_4_ 0.25 was used. Pre-culturing was performed for 18–20 h at the optimum temperature for the bacteria to achieve high viable cell counts.

Dual-species biofilms were formed in TSB broth with *L. monocytogenes* paired with *E. coli* or *S. aureus* at 37 °C, or with *P. putida* at 30 °C. Quantification of bacteria in the supernatants before and after treatments was done by spreading on selective media (Palcam for *L. monocytogenes*, Chromocult for *E. coli*, Pseudomonas Selective Agar for *P. putida*, and Baird Parker Agar for *S. aureus*). Lab M Limited (Heywood, UK) provided the first three media and Biolab (Budapest, Hungary) provided Baird Parker Agar.

### 2.2. EOs

EOs of cinnamon (*Cinnamomum zeylanicum*), marjoram (*Origanum majorana*), and thyme (*Thymus vulgaris*) were purchased from Aromax Natural Products Zrt. (Budapest, Hungary). Their main components, i.e., trans-cinnamaldehyde, terpinen-4-ol, and thymol, respectively, were obtained from Sigma-Aldrich (Munich, Germany). The composition of the oils was determined by GC-MS (Agilent GC: 6850 Series II; MS: 5975C VL MSD; Agilent, Santa Clara, CA, USA) using an Agilent 19091S-433E (Agilent) column at the laboratory of Aromax Natural Products Zrt.

### 2.3. Determination of Minimum Inhibitory Concentration (MIC)

For the determination of MIC values, the EOs and their components were diluted in liquid culture medium (TSB) in combination with Tween 40 (1%). At the concentration used, Tween 40 had no effect on the viability of the investigated bacteria.

One hundred microliters of 24 h-old cell suspension (10^6^ CFU/mL) of each bacterium in liquid culture medium was added to each well of a 96-well microtiter plate, followed by 100 μL of the diluted EO or its major component. Positive controls contained the inoculated growth medium without any EOs or components, and negative controls contained EOs or components in sterile medium. After 24 h of incubation at the appropriate temperature (37 °C for *E. coli*, *L. monocytogenes,* and *S. aureus* and 30 °C for *P. putida*), absorbance was measured at 600 nm (SPECTROstar Nano Spectrophotometer; BMG Labtech, Offenburg, Germany). Absorbance lower than 10% of the positive control samples, i.e., growth inhibition of 90% or more, was considered as the MIC value. Measurements were performed in triplicate.

### 2.4. Mono- and Dual-Species Biofilm Formation

For biofilm formation, the method described by Peeters et al. [33] was used. Briefly, polystyrene microtiter plates were inoculated with 200 μL of 24-h-old bacterial culture containing cell count of approximately 10^8^ CFU/mL. Following 4 h of cell adhesion at the corresponding temperatures, the supernatant was removed, and the plates were rinsed with physiological saline. Subsequently, 200 μL of fresh medium containing the EO or its component to be examined was added at MIC/2 concentration to avoid total growth inhibition. Plates were further incubated for 24 h to allow a biofilm to form. Positive controls contained the inoculated growth medium but without any EOs or components, and negative controls contained EOs or their components in growth medium. Experiments were repeated at least twice, and six parallel measurements were conducted each time.

The inhibition of biofilm formation was detected by the crystal violet staining method. Briefly, after 24 h of treatment, the supernatant was removed, and the wells were rinsed with physiological saline. For fixation of the biofilms, methanol was added, and the supernatant was removed again. Then, 0.1% crystal violet (CV) solution was added to each well and, 20 min later, the excess dye was removed by washing the plates under running tap water. The bound CV was released by adding 200 μL of 33% acetic acid followed by an incubation for 10 min at room temperature. The absorbance was measured at 590 nm (SPECTROstar Nano Spectrophotometer).

For polymicrobial biofilms, except for those produced from the culture containing *L. monocytogenes* and *E. coli*, 24-h-old liquid monocultures containing approximately 10^5^ CFU/mL were mixed at a 1:1 ratio in TSB broth. In contrast, the inoculum size for *L. monocytogenes* and *E. coli* was 10^3^ CFU/mL because the cell count of the dual culture reached more than 10^10^ CFU/mL after 24 h when a larger inoculum was used. For dual-species biofilms, the EO concentrations were chosen according to the MIC values of the individual strains. For investigation of the possible interactions, for example, synergism or antagonism between the species, concentrations corresponding to half and double the MIC level were examined. When the MIC values were equal for the two species, the levels corresponding to one-quarter of MIC, half of MIC, MIC, and double MIC were used.

### 2.5. Scanning Electron Microscopy (SEM) Observations

SEM was used to investigate the structural differences between biofilms before and after treatment with selected EOs and their components.

Polymicrobial biofilms were prepared in six-well microtiter plates, where sterile 2 × 2 cm cover slips served as the surfaces to which the cells attached. After 4 h of incubation, the cover slips were rinsed with sterile water, then, fresh medium containing the EOs or their components was added, and the plates were further incubated for 24 h at 37 °C. The concentrations of EOs and components used in this experiment were based on the results on dual-species biofilms.

Preparation of the cover slip-biofilm samples for SEM was performed as described previously [31]. Briefly, samples were immersed in 2.5% glutaraldehyde for 2 h at room temperature and then dehydrated using increasing ethanol concentrations. Final dehydration was performed with *t*-butanol–100% ethanol solution at different ratios, followed by absolute *t*-butanol. After replacing the *t*-butanol with a fresh volume of it and storing the samples at 4 °C for 1 h, they were freeze-dried overnight. Before SEM analysis, a gold membrane was applied, the whole field was examined, and photographs were taken from relevant areas with a Hitachi S4700 scanning electron microscope (Hitachi, Tokyo, Japan). Changes in the three-dimensional structure were mainly visualized with small scale magnification, while higher magnification was used for a more detailed view of the cell wall degrading effect of EOs.

### 2.6. Statistical Analysis

Data were analyzed by one-way ANOVA followed by Tukey’s test or paired *t*-test using GraphPad Prism version 6.0 (GraphPad Software Inc., San Diego, CA, USA. Differences were considered significant at *p* < 0.05.

## 3. Results

### 3.1. Composition of EOs and MICs

The compositions of the EOs were previously determined by the producers, and detailed data on them were also reported in previous studies [32,34]. The major components of the EOs were terpinen-4-ol (33.5%) for marjoram, trans-cinnamaldehyde (93.1%) for cinnamon, and thymol (51.8%) for thyme.

MIC values ranged between 0.25 and 20 mg/mL and the bacterium most sensitive to the agents was found to be *E. coli* (Table 1). Among the substances investigated, cinnamon and the components cinnamaldehyde and thymol presented the lowest MIC values. The latter result supports the generally accepted finding that phenolics have the best antimicrobial activity among EO compounds [25,35,36].

### 3.2. Anti-Biofilm-Forming Effect of EOs and Their Major Components

#### 3.2.1. Monocultures

Table 2 summarizes the effects of the tested EOs on monoculture biofilms. For *E. coli*, each components and the thyme EO showed significant inhibitory effect when compared to the control. Cinnamon, despite its notable antibacterial effect, did not reduce the biofilm-forming ability of *E. coli*, but its main component and thyme did. Thymol exhibited the best effect against biofilm formation.

All EOs and their investigated components had considerable anti-biofilm-forming effects on *L. monocytogenes*, as reflected in significant differences compared with the control. Cinnamon and trans-cinnamaldehyde were the best inhibitors and there were no significant differences between the anti-biofilm-forming capacity of the EOs and their main components (*p* > 0.05). Similar results could be seen in the case of *P. putida*, for which cinnamon, thyme, and their major components inhibited biofilm formation, but no significant differences were observed among these groups. Marjoram and terpinene-4-ol also had a strong effect against biofilm formation of all bacteria studied.

For *S. aureus* biofilms, all EOs and components tested proved to be effective against their formation. Absorbance of the treated samples differed significantly from that in the control (*p* < 0.001), but not from each other (Table 2).

#### 3.2.2. Polymicrobial Cultures

##### *L. monocytogenes* and *E. coli*

**Figure 1:**
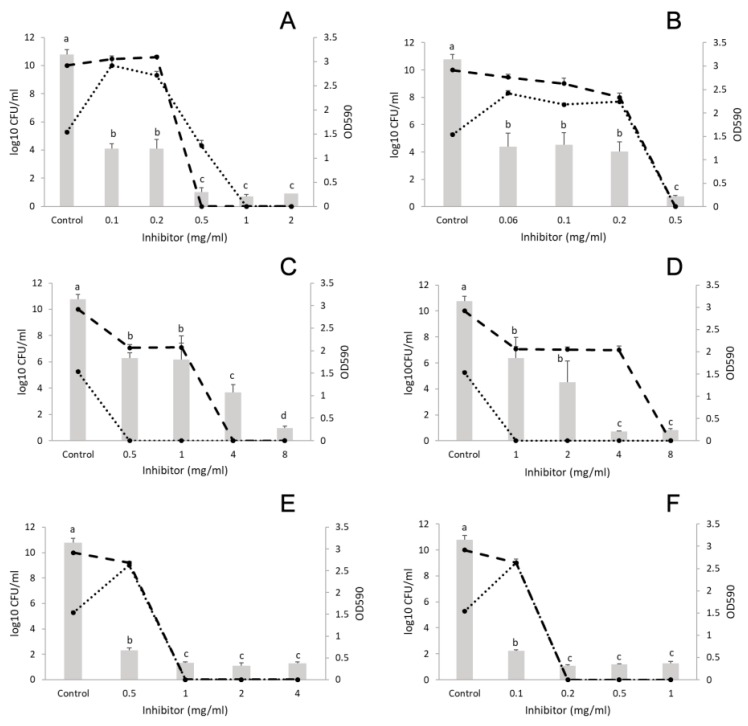
Effect of essential oils (EOs) on the biofilm formation of *Listeria monocytogenes* and *Escherichia coli* polymicrobial cultures. (**A**) cinnamon EO, (**B**) cinnamaldehyde, (**C**) marjoram EO, (**D**) terpinen-4-ol, (**E**) thyme EO, and (**F**) thymol. Columns represent the OD_590_ values, dashed lines represent cell numbers of *L. monocytogenes*, and dotted lines cell numbers of *E. coli*. Results are presented as mean ± standard deviation of six replicates. Different letters indicate statistically significant differences between columns (*p* < 0.05).

The concentration used for cinnamon was 0.1–2 mg/mL and for trans-cinnamaldehyde 0.06–0.5 mg/mL. After biofilm formation for 24 h, the cell number of control samples was 11 log CFU/mL for *L. monocytogenes* and 5 log CFU/mL for *E. coli*. Treatment with cinnamon at concentrations higher than 0.1 mg/mL decreased the biofilm formation (CFU: 10 log CFU/mL for both bacteria) (Figure 1A). At 0.25 mg/mL (MIC of *E. coli*), both bacteria grew up to 10 log CFU/mL each. At 0.5 mg/mL concentration (double the MIC for *E. coli* and half the MIC for *L. monocytogenes*, see Table 1), a monoculture biofilm appeared with *Listeria* being absent. From a concentration of 1 mg/mL (MIC for *L. monocytogenes*, see Table 1), no survivors of either bacterium were detected. Trans-cinnamaldehyde exhibited similar activity against biofilm formation as compared to cinnamon (Figure 1B).

After treatment with marjoram (concentration range: 0.5–8 mg/mL) and the component terpinen-4-ol (concentration range: 1–8 mg/mL), cell number analysis showed that *E. coli* was eliminated from the biofilm at the lowest concentration, so that this culture contained *L. monocytogenes* cells only (*p* < 0.001). Biofilm elimination started form the lowest concentrations used (0.5 mg/mL for marjoram and 1 mg/mL for terpinene-4-ol) (Figure 1C,D).

Finally, thyme EO (concentration range: 0.5–4 mg/mL) exhibited strong inhibitory effect against the *L. monocytogenes* and *E. coli* mixed culture biofilm, at all the concentrations investigated (*p* = 0.03) (Figure 1E). Moreover, no survivors were detected at 1 mg/mL (half the MIC for *L. monocytogenes*, see Table 1). In addition, thymol (concentration range: 0.1–1 mg/mL) inhibited biofilm formation from its lowest concentration (Figure 1F). Surviving cells were undetectable at the sub-MIC value of 0.2 mg/mL.

##### *L. monocytogenes* and *S. aureus*

**Figure 2:**
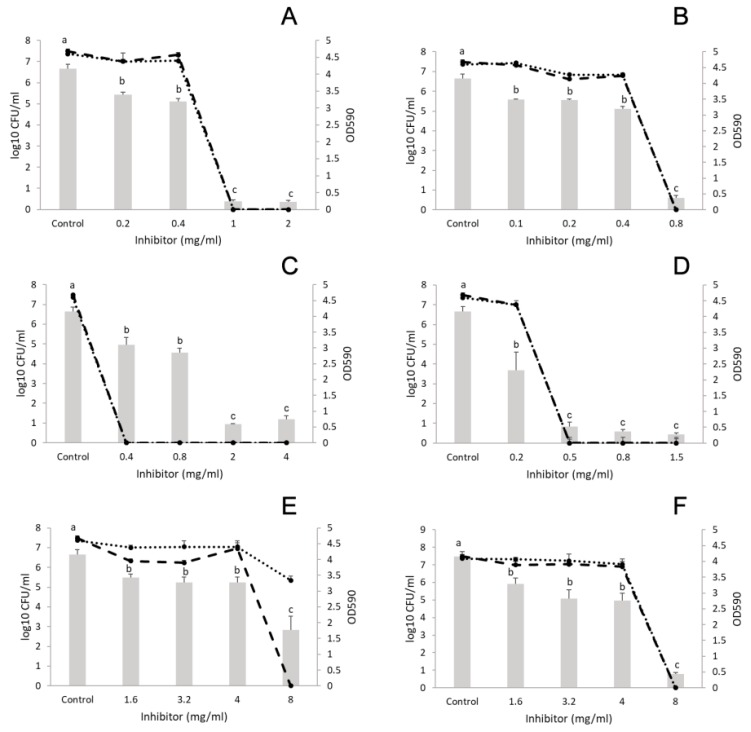
Effect of EOs on the biofilm formation of *Listeria monocytogenes* and *Staphylococcus aureus* polymicrobial cultures. (**A**) cinnamon EO, (**B**) trans-cinnamaldehyde, (**C**) thyme EO, (**D**) thymol, (**E**) marjoram EO, and (**F**) terpinen-4-ol. Columns represent the OD_590_ values, dashed lines represent cell numbers of *L. monocytogenes*, and dotted lines cell numbers of *S. aureus*. Results are presented as mean ± standard deviation of six replicates. Different letters indicate statistically significant differences between columns (*p* < 0.05).

In the control sample, 7 log CFU/mL was recorded for both bacteria after biofilm formation for 24 h. All EOs and their components significantly decreased biofilm formation (Figure 2). The results indicate that only concentrations higher than the MIC values were effective at eliminating the bacteria. At 8 mg/mL (double the MIC for *Listeria* and more than double the MIC for *S. aureus*, see Table 1), marjoram EO (concentration range: 1.6–8 mg/mL) eliminated *Listeria* from the mixed culture, but *S. aureus* was still present (Figure 2E). At the same concentration, terpinen-4-ol (concentration range: 1.6–8 mg/mL) reduced the CFU of both bacteria to an undetectable level (Figure 2F). Similar results were obtained with cinnamon (concentration range: 0.2–2 mg/mL) and its component trans-cinnamaldehyde (concentration range: 0.1–0.8 mg/mL) (Figure 2A,B). Moreover, thyme (concentration range: 0.4–4 mg/mL) reduced polymicrobial biofilms from a concentration of 0.4 mg/mL (MIC for *L. monocytogenes*) (Figure 2C). Finally, thymol (concentration range: 0.2–1.5 mg/mL) reduced the biofilm investigated and killed both bacteria from a concentration of 0.2 mg/mL (MIC for *Listeria*) (Figure 2D).

##### *L. monocytogenes* and *P. putida*

**Figure 3:**
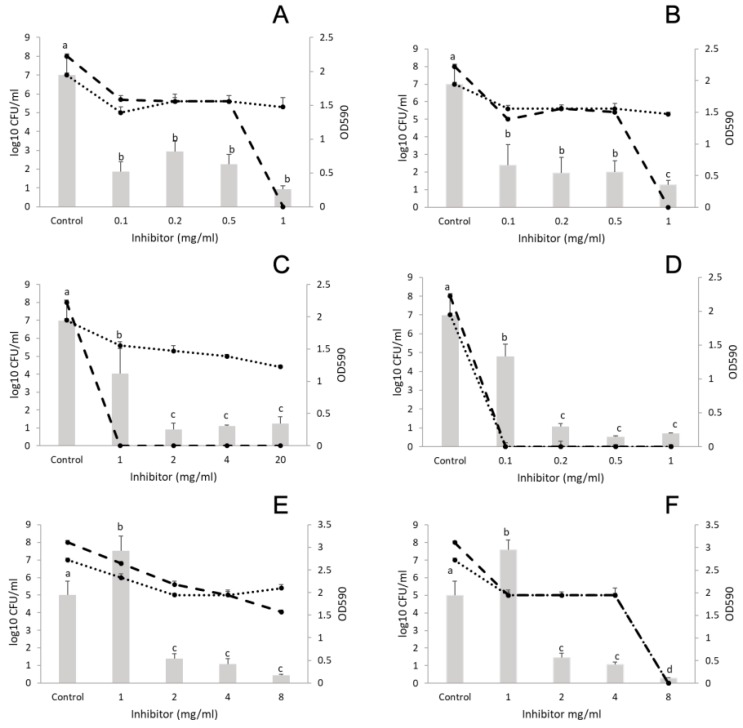
Effect of EOs and EO main components on the biofilm formation of *Listeria monocytogenes* and *Pseudomonas putida* polymicrobial cultures. (**A**) cinnamon EO, (**B**) trans-cinnamaldehyde, (**C**) thyme EO, (**D**) thymol, (**E**) marjoram EO, and (**F**) terpinen-4-ol. Columns represent the OD_590_ values, dashed lines represent cell numbers of *L. monocytogenes*, and dotted lines cell numbers of *P. putida*. Results are presented as mean ± standard deviation of six replicates. Different letters indicate statistically significant differences between columns (*p* < 0.05).

Figure 3 shows that the number of *Listeria* cells was 8 log CFU/mL and that of *P. putida* was 7 log CFU/mL in the control samples. Concerning cinnamon EO (concentration range: 0.1–1 mg/mL) and trans-cinnamaldehyde (concentration range: 0.1–1 mg/mL), significant reduction in biofilm formation was observed at 0.1 mg/mL; although higher concentrations up to 0.5 mg/mL did not achieve better inhibition (Figure 3A, B). Thyme EO (concentration range 1–20 mg/mL) inhibited the formation of *L. monocytogenes* and *P. putida* co-cultured biofilm as well, at 1 mg/mL (Figure 3C). However, *P. putida* was present (4 log CFU/mL) in the biofilm even at the highest concentration of thyme (20 mg/mL) (Figure 3C), which was equal to the MIC against this bacterium (Table 1). Thymol was applied between 0.1–1 mg/L where biofilm inhibition and decrease in CFU started at the lowest concentration used.

Marjoram EO (concentration range: 1–8 mg/mL) decreased the number of *P. putida* cells at 8 mg/mL by only 2 log CFU/mL compared with the control (double the MIC for *Listeria* and four times the MIC for *P. putida*), (Figure 3E). Meanwhile terpinene-4-ol (concentration range: 1–8 mg/mL) eliminated both bacteria from the biofilm at 8 mg/mL and had similar biofilm inhibitory effect to the parent oil.

### 3.3. SEM Observations

#### 3.3.1. *L. monocytogenes* and *E. coli*

Cinnamon and trans-cinnamaldehyde were not used in these investigations due to their high inhibitor effect. For *Listeria* and *E. coli* only thyme EO and thymol were chosen based on results obtained for dual-species biofilm.

**Figure 4:**
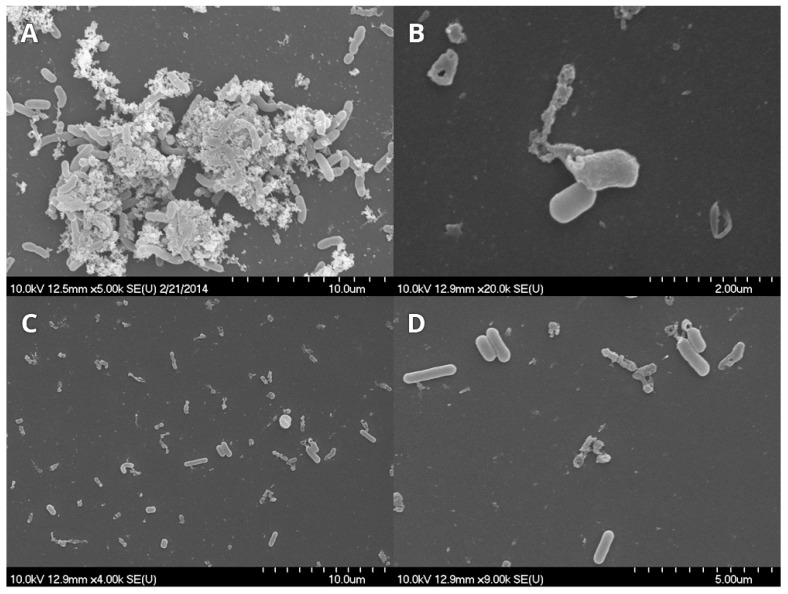
*Listeria monocytogenes* and *Escherichia coli* biofilms observed using scanning electron microscope. (**A**) control, (**B**) thyme EOs (0.5 mg/mL), and (**C**,**D**) thymol (0.1 mg/mL).

Control samples showed the complex structure of the formed biofilm (Figure 4A), namely, cells embedded in a large amount of possible extracellular polysaccharide (EPS) and forming a three-dimensional structure. Treatment with thyme and thymol resulted in damaged, anamorph, sparse micro-colonies and individual cells (Figure 4B–D).

#### 3.3.2. *L. monocytogenes* and *S. aureus*

**Figure 5:**
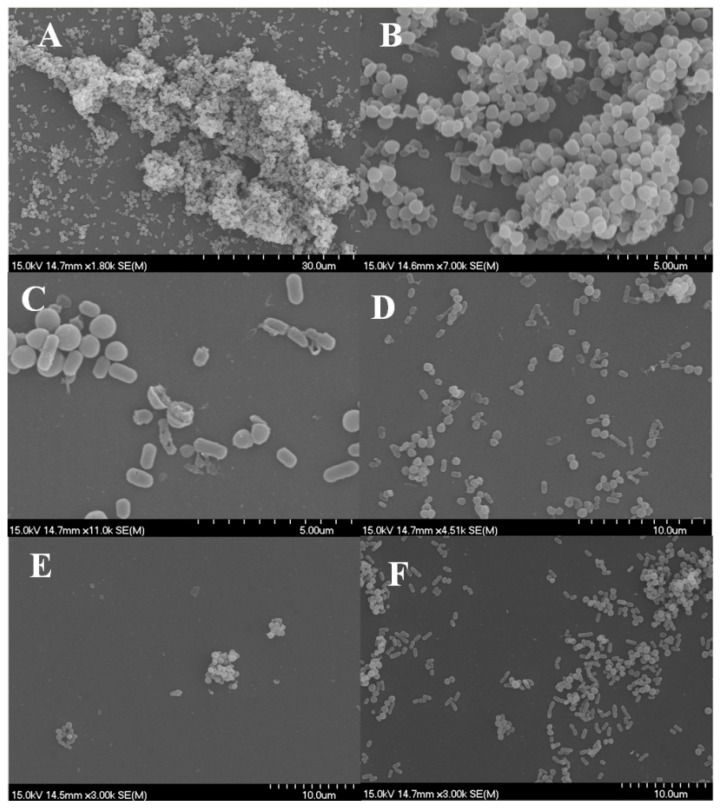
*Listeria monocytogenes* and *Staphylococcus aureus* biofilms observed using scanning electron microscope. (**A**,**B**) control, (**C**) marjoram essential oil (EO) (1.6 mg/mL), (**D**) terpinen-4-ol (8 mg/mL), (**E**) thyme EO (0.4 mg/mL), and (**F**) thymol (0.8 mg/mL).

Figure 5A,B present the findings for control samples of *Listeria* and *Staphylococcus* polymicrobial biofilms. The three-dimensional structure embedded in the EPS layer can be observed with *Listeria* cells are at the bottom, to which *Staphylococcus* clusters attach. After treatment with EOs, this structure appears only in micro-colonies with a simple shape, the number of attached cells is decreased, and mainly damaged cells are present. Samples treated with marjoram show split cocci, in agreement with the fact that the EO’s target is the cell wall. In some cases, cell debris appears on the surface, which can serve as a possible adhesion site for new cells (Figure 5C–F).

#### 3.3.3. *L. monocytogenes* and *P. putida*

For *Listeria* and *P. putida* only thyme EO and thymol were chosen based on results obtained for dual-species biofilm.

**Figure 6:**
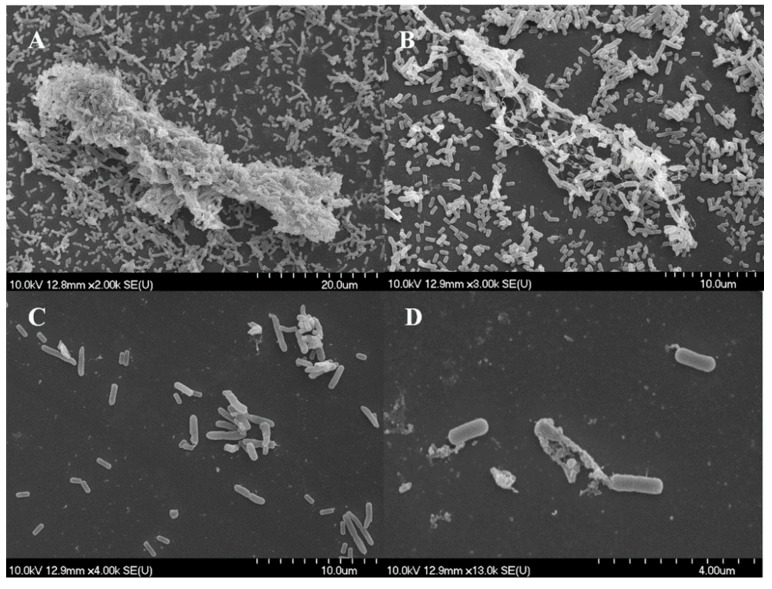
*Listeria monocytogenes* and *Pseudomonas putida* biofilms observed using scanning electron microscope. (**A**,**B**) control, (**C**) marjoram EO (1 mg/mL), and (**D**) terpinen-4-ol (1 mg/mL).

Figure 6A,B present the findings for control samples with nascent and fully formed cell-to-cell connections and EPS material formation. Treatment with marjoram and its major component, terpinen-4-ol, resulted in significant changes in the composition of these structures, leaving mainly damaged cells and cell debris to be observed (Figure 6C,D).

Scanning electron microscopy images demonstrate the structural changes caused by EOs or components during biofilm development [36]. The three-dimensional structure of matured biofilms disappeared after EO treatment, the cells were damaged, and most of the treated cells had burst.

## 4. Discussion

In the present study we demonstrated that cinnamon, marjoram, and thyme EOs and main components tested had good antibacterial and anti-biofilm forming effect on the investigated bacteria associated with food spoilage and outbreaks. Trans-cinnamaldehyde and thymol were the best inhibitors with MIC values below 1 mg/mL. As expected from the composition pattern of EO cinnamon, the trans-cinnamaldehyde and the EO exhibited similar effect against the bacterial biofilms studied. Besides this, thymol and trans-cinnamaldehyde are phenolics having the best antimicrobial activity among the EO compounds. For comparison, the monoterpene terpiene-4-ol did not exceed the effect of its parent oil marjoram.

Monoculture biofilms were significantly inhibited by the EOs and components in MIC/2 concentration which suggest that growth-reducing effect is not solely responsible for biofilm inhibition. Sub-lethal damage of the cell wall can negatively influence bacterial attachment to surfaces which is the first step in biofilm formation [29].

Dual-species biofilms responded to EOs differently. Contrary to our findings, Almeida et al. [37] reported that, in dual-species (*E. coli*/*L. monocytogenes*) biofilms, cell densities were not altered. In our case, *L. monocytogenes* outgrew *E. coli* in the control samples and most of the EOs eliminated this bacterium from the biofilm. In line with this, the study of Giaouris et al. [9] concluded that *Listeria* formed a stronger biofilm in mixed population, moreover, presence of other bacteria increased its growth. Co-culturing of *L. monocytogenes* and *S. aureus* resulted a strong biofilm, which is in agreement with the results of Millezi et al. [38]; only high concentrations of EOs (e.g., cinnamon EO: 1–2 mg/mL, marjoram EO, and terpinene: 4–8 mg/mL) and components inhibited their formation. *P. putida* proved to be more resistant to the oils and compounds than the *Listeria* and this bacterium was present in the population even at high agent concentrations. This is in accordance with the results of Giaouris et al. [9] who showed that co-culturing with *L. monocytogenes* within a dual-species biofilm increased the community resistance of *P. putida*.

In most cases, anti-biofilm formation effect showed no concentration dependence above a certain concentration, but the results of cell number determination were not congruent with this. In most cases, dual-species biofilms were inhibited at lower concentration than the MIC of the individual bacteria, but cell death occurred mainly at the higher MIC value or above. This discrepancy pointed to the fact that absorbance and cell enumeration data can differ significantly. Biofilms, inhibited to a high degree based on absorbance data, can still contain enough living cells to cause hygiene problems. Scanning electron microscopic images demonstrated the structural changes caused by EOs or components during biofilm development. Similarly to the altered biofilm structure visualized by confocal laser scanning electron microscope in a recent study [39], our SEM pictures also showed that the three-dimensional structure of matured biofilms disappeared after EO treatment and most of the treated cells have been burst. Studying the mechanism of EOs in more detail, Zhang et al. [40] detected leakage of electrolytes due to disruption of cell permeability after EO treatment which eventually lead to cell death. Along with our findings these results also support the fact that EOs induce severe membrane damage [25,40].

In conclusion, the EOs and EO components examined in this study could represent alternatives for elimination of *E. coli*, *L. monocytogenes*, *P. putida,* and *S. aureus* single and *L. monocytogenes*-*E. coli*/*S. aureus*/*P. putida* dual biofilms in vitro with different efficiency. The use of EOs as antimicrobial agents in real food systems is often limited due to their strong odor and taste. Therefore, our future investigations aim at novel approaches, such as encapsulation of EOs, that could potentially reduce the organoleptic impact and increase the antimicrobial activity [41].

## Figures and Tables

**Table 1:** Minimum inhibitory concentration (MIC) of essential oils (EOs) and their components against the food-related bacteria investigated.

EOs and Components	MIC (mg/mL)			
	*Escherichia coli*	*Listeria monocytogenes*	*Pseudomonas putida*	*Staphylococcus aureus*
Cinnamon	0.25	1	0.25	0.4
Marjoram	0.5 ^1^	4 ^2^	2 ^1^	3.2
Thyme	1.0	2 ^2^	20	0.8
Trans-cinnamaldehyde	0.25	0.25	0.25	0.4
Terpinene-4-ol	2 ^1^	4	4 ^1^	3.2
Thymol	0.5	0.5 ^2^	0.5	0.8

^1^ Results from Kerekes et al. (2013). ^2^ Results from Kerekes et al. (2016).

**Table 2:** Effect of essential oils and components (in MIC/2 concentration) on monoculture biofilm formation of food-related bacteria.

Bacteria	Biofilm Formation (OD _590_) ^1^						
	Positive Control	Cinnamon	Marjoram	Thyme	Trans-cinnamaldehyde	Terpinene-4-ol	Thymol
*Escherichia coli*	0.68 ± 0.17a	0.77 ± 0.13a	0.72 ± 0.10a ^2^	0.34 ± 0.02b	0.32 ± 0.08b	0.44 ± 0.11b ^2^	0.14 ± 0.02c
*Listeria monocytogenes*	1.34 ± 0.14a	0.14 ± 0.02c	0.28 ± 0.04b	0.30 ± 0.04b	0.17 ± 0.02c	0.34 ± 0.34b	0.53 ± 0.08b
*Pseudomonas putida*	1.52 ± 0.4a	0.48 ± 0.05d	0.08 ± 0.00b ^2^	0.51 ± 0.05d	0.70 ± 0.05d	1.09 ± 0.10b ^2^	0.43 ± 0.07d
*Staphylococcus aureus*	2.36 ± 0.07a	1.31 ± 0.30b	1.18 ± 0.20b	1.22 ± 0.16b	1.19 ± 0.17b	1.60 ± 0.30b	1.37 ± 0.41b

^1^ Data are presented as mean ± standard deviation of six replicates. Values within a row with different letters are significantly different (*p* < 0.05). ^2^ Results from Kerekes et al. (2013).
